# Performance of Portfolios Based on the Expected Utility-Entropy Fund Rating Approach †

**DOI:** 10.3390/e23040481

**Published:** 2021-04-18

**Authors:** Daniel Chiew, Judy Qiu, Sirimon Treepongkaruna, Jiping Yang, Chenxiao Shi

**Affiliations:** 1 Business School, The University of Western Australia, Perth 6009, Australia; Daniel.chiew@msn.com (D.C.); uwajqiu@gmail.com (J.Q.); sirimon.treepongkaruna@uwa.edu.au (S.T.); 2 School of Economics and Management, Beihang University, Beijing 100083, China; beihang_chenxiao@163.com

**Keywords:** expected utility–entropy, fund rating, risk, portfolio, performance

## Abstract

Yang and Qiu proposed and reframed an expected utility–entropy (EU-E) based decision model. Later on, a similar numerical representation for a risky choice was axiomatically developed by Luce et al. under the condition of segregation. Recently, we established a fund rating approach based on the EU-E decision model and Morningstar ratings. In this paper, we apply the approach to US mutual funds and construct portfolios using the best rating funds. Furthermore, we evaluate the performance of the fund ratings based on the EU-E decision model against Morningstar ratings by examining the performance of the three models in portfolio selection. The conclusions show that portfolios constructed using the ratings based on the EU-E models with moderate tradeoff coefficients perform better than those constructed using Morningstar. The conclusion is robust to different rebalancing intervals.

## 1. Introduction

Mutual funds have become an increasingly dominant choice for retail investors in recent years, underlined by the large number of investors who attempt to beat the market and those who seek to diversify away the unsystematic risk from their portfolio [1,2]. In selecting funds to comprise their portfolios, investors would seek to invest in the best performing funds. As a result, investors would explore the best rating category to guide their investment decisions. At present, the most prominent fund rating approaches have been developed by agencies, including Morningstar and Lippers. These approaches rank funds using a star rating system on a scale of 5 to 1, where 5 stars are deemed to be the “best” performing funds, based on a fund’s calculated risk-adjusted return. Studies have shown Morningstar ratings play a powerful role in the mutual fund industry, seen as a crucial metric for investors and fund managers [3,4]. However, Sharpe [5], Lisi and Caporin [6] showed that risk adjustment made in calculating fund rating in Morningstar may not account for the risk faced by a fund appropriately. This inefficient adjustment for risk may be attributed, in part, to Morningstar’s reliance on the expected utility theory, which is proven to draw conclusions which deviate from individuals’ behavior under risk [7,8,9]. Specifically, Kahneman and Tversky [8] pointed out that people deviate from the description of expected utility theory in actual decision-making. Since its descriptive power for risky choices has been challenged and discussed by some famous paradoxes and researchers [7,10,11], many alternatives models have been developed to provide additional insights about decision-making under risk [8,12,13,14,15,16,17], especially the decision-making models to expand expected utility involving Shannon entropy [18,19,20,21,22,23]. Shannon entropy [24] has been applied to a wide body of financial literature to guide investors’ investment decisions for its ability to describe risks. Recent studies evaluating entropy relative to standard deviation and beta in measuring financial risk have supported entropy, primarily for its distribution-free nature and ability to incorporate more information of uncertainty than the latter two measures [19,25]. The conclusions of Bentes and Menezes [26] show that entropy can more clearly indicate market volatility. Bentes et al. [27] investigate the volatility characteristics of the stock market returns and compare the conditionally heteroscedastic models with Shannon entropy, Renyi entropy and Tsallis entropy. They conclude that entropy can be helpful due to its advantage in capturing the uncertainty of the stock returns without any constraints on the probability distribution of the return series. Caraiani [28] found that entropy can predict dynamic changes in the market, showing that entropy has important characteristics in predicting fund performance. Furthermore, there are some recent studies about entropy to measure uncertainty applied to decision-making. Wei et al. [29] proposed a general form of entropy measures for hesitant fuzzy sets. Wei et al. [30] further investigated how to measure the uncertainty of hesitant fuzzy linguistic term sets and extended hesitant fuzzy linguistic term sets and then applied the measures to compute the weights in decision-making problems.

As expected utility cannot be a descriptive decision-making model, and entropy alone is unable to consider the outcome of the risky action. Yang and Qiu [22] proposed the expected utility–entropy (EU-E) decision model. The EU-E model incorporates the notion of expected utility and entropy together to establish a decision model, which effectively considers the decision maker’s subjective preference and objective uncertainty at each state of nature. Additionally, this model is proven to solve typical decision problems, such as the Allais paradox reasonably, which the expected utility theory is known to be incapable of. As it has previously been argued that investors’ behaviors differ to the constraints set by the axioms of the expected utility theory, the finding that the EU-E model can solve these decision problems indicates the consideration of a behavioral component inherent in the model [31].

The EU-E decision model is not established on an axiomatic basis. Luce et al. [21] developed the numerical representations for risky actions under behavioral axioms similar to the EU-E decision model in Yang and Qiu [22]. Furthermore, Yang and Qiu [23] improved the model to a normalized EU-E decision model, allowing for comparison of risky choices where the numbers of states are widely dispersed. Dong et al. [32], Xia et al. [33], and Xia et al. [34] presented several discussions on the EU-E decision model and emphasizing the role of Shannon entropy in the field of decision-making under risk. Casquilho and Rego [35] used decision models with different utility values combined with weighted entropies, respectively, incorporating rarity factors associated with Gini-Simpson and Shannon measures. In their paper, they provided an example of this decision framework for landscape compositional scenarios in Portugal. Their results indicate that the likely best combination is achieved by the criterion using the Shannon weighted entropy and a square root utility function. Allahverdyan et al. [36] derived a measure of risk similar to the EU-E measure of risk and concluded that their risk measure has normative features. The EU-E model also has been applied in other fields, such as a decision-making model for large consumers on a smart grid [37] and in rainfall threshold analysis [38].

In addition, Yang et al. [39] applied the EU-E decision model to stock selection using different tradeoff coefficients within certain intervals to derive efficient portfolios with respect to the traditional mean-variance framework. They found that the efficient portfolios from stocks selected using the EU-E model with intermediate values of tradeoff coefficients are more efficient. Thus, the intermediate values of tradeoff coefficients in the EU-E model are more reasonable. The conclusions in their paper demonstrate the applicability of the EU-E decision model.

Chiew et al. [40] applied the EU-E decision model to fund ratings and proposed an alternative fund rating approach based on the EU-E decision model. We applied this approach to mutual fund ratings in the US market and examined the predictive ability of this approach for its ability to potentially mitigate the drawbacks of the risk measure used in Morningstar ratings. In the paper, the ratings based on the EU-E model have been compared with longstanding fund rating measure, Morningstar ratings, across a 13 year in-sample period. We found that ratings based on the EU-E model where the tradeoff coefficient takes values of 0.25 and 0.75 outperform Morningstar in predicting the best but not the worst-performing funds. This result is robust to varying market climates.

As we demonstrated that the fund rating approach based on the EU-E model could predict the best performing funds [40], it raised the question of whether investors can utilize this fund rating approach to guide their investment decisions and then achieve excess returns. This motived us to investigate whether the established EU-E fund rating approach is helpful in guiding investors to make investment decisions.

To further investigate the utilization of the EU-E fund rating approach to guide investment decisions, we constructed portfolios using the best ranking funds based on the established rating approach and then evaluated the portfolios performance. To examine the performance of portfolios constructed based on the EU-E fund rating approach, we evaluated the performance of portfolios based on the established rating approach against Morningstar ratings. Specifically, we investigated the performance of portfolios constructed based on the best rating funds by randomly selecting 15 funds from the 5-star category ranked both by fund ratings based on the EU-E model and Morningstar. We have shown that the fund rating approach is practical for investors to construct fund portfolios by selecting the best performing funds.

## 2. A Fund Rating Approach Based on the EU-E Decision Model

### 2.1. A Fund Rating Approach Based on the EU-E Decision Model

To apply the fund rating approach based both on the EU-E decision model and Morningstar ratings [40] to constructing portfolios from the best performing funds and then investigate the performance of these portfolios, we first introduce this fund rating approach in this section. The definitions of the EU-E measure of risk are first shown in Definition 1 as follows [22,23].

**Definition** **1.** *Given a general decision analysis model* G=(Θ,A,U) *, action* a∈A *, state of nature* θ∈Θ *. Suppose there exists at least two actions in the action space and at least two states in the state space, respectively, and the decision-maker’s utility function u(x) is mono-increasing. If* $\max_{a\in A}\{|E[u(X(a,\theta ))]|\}$ *is nonzero, the EU-E measure of risk of a risky action a is defined as follows:*

(1) $$R(a)=\lambda \frac{H_{a}(\theta )}{\ln(n_{a})}-(1-\lambda )\frac{E[u(X(a,\theta ))]}{\max_{a\in A}\{\left|E[u(X(a,\theta ))]\right|\}}$$

*where λ is the tradeoff coefficient, reflecting a tradeoff between the decision-maker’s subjective expected utility and objective uncertainty of an action. It takes the value from 0 to 1.* $n_{a}$ *is the number of states corresponding to action a,* $H_{a}(\theta )$ *denotes the Shannon entropy of the distribution of its corresponding states;* X(a,θ) *denotes the outcome corresponding to state θ when taking action a,* E[u(a)] *is the expected utility of action a.*

Investing in funds is a risk decision-making problem. Given a set of funds aiming at ranking the funds according to the above-defined EU-E measure of risk, the following fund rating approach is established.

An investor wants to select a set of funds to compose portfolios. $S=\{S_{1},S_{2},\cdots ,S_{m}\}$ is the set of funds, $A=\{a_{1},a_{2},\cdots ,a_{m}\}$ is the action space, and $a_{i}$ is to select fund $S_{i}$ (*i* = 1, 2, …, *m*). $R_{i}=\{r_{i1},r_{i2},\cdots ,r_{il}\}$ is the set of returns of *l* previous months. Let $a=\underset{1\leq i\leq m}{\min}\{r_{i1},r_{i2},\cdots ,r_{il}\}$, $b=\underset{1\leq i\leq m}{\max}\{r_{i1},r_{i2},\cdots ,r_{il}\}$. We create the distribution of monthly returns by dividing interval [a, b] into *n* equal subintervals $\left[r_{0},r_{1}\right)$, $\left[r_{1},r_{2}\right)$, …, $\left[r_{n-1},r_{n}\right]$. Let $\theta_{j}$ denote these intervals, respectively. Then, $\Theta_{i}=\{\theta_{i1},\theta_{i2},\cdots ,\theta_{in_{i}}\}$ is the state space, corresponding to action $a_{i}$. The decision model for fund investment can be summarized as G=(Θ,A,u), and *u*(*x*) is the investor’s utility function.

We then calculate the frequency of fund $S_{i}$’s return, which falls within interval $\theta_{j}$, denoted by $\rho_{ij}$, and let the expected return in interval $\theta_{j}$ of $S_{i}$ be ${\bar{x}}_{ij}$. According to Bernoulli’s law of large numbers, $\rho_{ij}$ approaches the probability of the fund $S_{i}$, $p_{ij}$, as *l* increases. Thus, this forms the probability distribution for each fund at each particular month. Consequently, we can assume that in the future, the return of fund $S_{i}$ will take an expected value of ${\bar{x}}_{ij}$ within interval $\theta_{j}$ drawn from the probability distribution, $p_{ij}$.

From the probability distribution, we can then calculate the Shannon entropy of state of nature corresponding to risky action $a_{i}$ as:

(2) $$H_{a_{i}}(\theta )=-\sum_{j=1}^{n_{i}}\rho_{ij}\ln\rho_{ij}$$

According to Definition 1, the EU-E measure of risk of investing in fund $S_{i}$ is shown in Equation (3) as follows:

(3) $$R(a_{i})=-\lambda \frac{\sum_{j=1}^{n_{i}}\rho_{ij}\ln\rho_{ij}}{\ln(n_{i})}-(1-\lambda )\frac{\sum_{j=1}^{n_{i}}\left[u({\bar{x}}_{ij}\right)\rho_{ij}]}{\underset{1\leq i\leq m}{\max}\{|\sum_{j=1}^{n_{i}}u({\bar{x}}_{ij})\rho_{ij}|\}}$$

where $R(a_{i})$ is the risk measure of investing in fund $S_{i}$ (*i* = 1, 2, …, *m*).

Next, we define the net normalized expected utility yielded by each fund in Equation (4). That is, we calculate each fund’s net normalized expected utility for each month by taking a fund’s risk from its normalized expected utility.

(4) $$Net\;E[u(a_{i})]=\frac{\sum_{j=1}^{n}\left[u({\bar{x}}_{ij})p_{ij}\right]}{\underset{1\leq i\leq m}{\max}\left\{\left|\sum_{j=1}^{n}\left[u({\bar{x}}_{ij})p_{ij}\right]\right|\right\}}-R(a_{i})$$

The net normalized expected utility is the EU-E risk adjusted expected utility, which considers both the expected utility and the EU-E risk metric. The net normalized expected utility can reflect investors’ preference better when the decision-maker considers the uncertainty of the action plan. For action $a_{1}$ and $a_{2}$, if $Net\;E[u(a_{1})]>Net\;E[u(a_{2})]$, then the action $a_{1}$ is preferred than the action $a_{2}$, that is, the higher the net expected utility, the better the action. We then rank all the actions in action space by the net expected utility in ascending order, where an action with a higher value is preferred.

Finally, the funds are categorized into ratings of 1 to 5 stars using the same quantile as the Morningstar rating, that is, the top 10% of the funds are rated 5 stars; the next 22.5% are rated 4 stars; the next 35% are rated 3 stars; the following 22.5% are rated 2 stars, and the last 10% are rated 1 star. We apply the above procedure to the funds’ past 3-, 5- and 10-year monthly returns to obtain their respective 3-, 5- and 10-year ratings throughout the sample. Next, we obtain each fund’s overall rating using Morningstar’s “50:30:20” approach each month, resulting in the monthly fund rankings based on the EU-E model [40,41].

It should be noted that existing literature does not specify a specific λ value, which provides the most efficient trade-off between expected utility and Shannon entropy in measuring risk. Furthermore, it is important to distinguish that γ used by Morningstar is not equivalent to λ in the EU-E decision model. γ refers to the degree of risk-aversion under the expected utility theory and defines the shape of the utility curve, whereas λ refers to the relative weighting of entropy to expected utility and does not determine the utility curve in the EU-E model.

### 2.2. Portfolios and Their Performance Constructed Using EU-E Model and Morningstar Ratings

In this section, we construct portfolios using both the EU-E model and Morningstar ratings. We assume that investors utilize fund ratings to guide their investment decisions, and they only take into account the best-performing funds to construct their portfolios. For this analysis, we assume that investors seek to invest in the highest-rated funds. As such only 5-star funds are used to construct the portfolios.

Alexeev and Dungey [42] demonstrate that an investor is able todiversify away the majority of unsystematic risk by investing in 6–15 stocks. Thus, we use 15 funds in constructing each portfolio. At first, we construct *n* equally weighted portfolios by randomly selecting 15 funds from the 5-star category ranked by Morningstar. Similarly, other *n* equally weighted portfolios are constructed based on the 5-star funds ranked by each of the EU-E models.

Suppose we rebalance the portfolios after *m* months, i.e., the rebalancing interval is m months. This means if a fund has lost its previous 5-star rating at the time of rebalancing, it will be replaced by a randomly selected fund from the 5-star category. We hold the portfolios for *T* intervals. We evaluate the performance of portfolios constructed using Morningstar ratings and ratings based on the models, respectively.

We present the methods used to evaluate the performance of portfolios following Loviscek and Jordan [43]. First, we calculate the geometric mean of the excess returns relative to the benchmark for each portfolio at every rebalancing interval. Next, we conduct the Wilcoxon signed-ranks test for differences between the performance of the Morningstar and EU-E model based portfolios relative to the benchmark. As shown by Loviscek and Jordan [43], the Wilcoxon signed-ranks test is used as opposed to a parametric test due to a potential bias associated with a low number of observations and anon-normal distribution.

In addition, we also assess the ability of an investor to construct trading strategies using the ratings based on Morningstar and the EU-E model to generate positive abnormal returns [44,45]. Furthermore, it is a usual practice to measure and test the performance of security returns to the security-specific event using the abnormal return (AR), average abnormal return (AAR), cumulative average abnormal return (CAAR) and t-test statistic [45,46,47,48,49]. In this paper, we take the strategy to construct portfolios as the security-specific event and use these measures of the portfolios performance and t-test statistic in the above reference. To do so, we calculate the cumulative average abnormal returns of the *n* portfolios constructed from 5-star funds based on either Morningstar or the EU-E models at every rebalancing interval.

The abnormal return of portfolio *i* for a particular rebalancing interval is defined as follows:

(5) $$AR_{it}=R_{it}-R_{mt}$$

where $R_{it}$ is the return of portfolio *i* at rebalancing interval *t* and $R_{mt}$ is the return of the benchmark at rebalancing interval *t*.

Therefore, we can define the average abnormal returns (AAR) for the *n* portfolios at each rebalancing interval *t* as:

(6) $$AAR_{t}=\frac{1}{n}\sum_{i=1}^{n}AR_{it}$$

Thus, the cumulative average abnormal returns (CAAR) of the *n* portfolios created from the beginning of the overall sample period to rebalancing interval *T* is:

(7) $$CAAR_{T}=\sum_{t=0}^{T}AAR_{t}$$

The significance of the CAARs is tested using *t*-statistics as follows:

(8) $$t_{CAAR}=\frac{CAAR_{T}}{\sigma_{CAAR}/\sqrt{T}}$$

where $\sigma_{CAAR}$ is the standard deviation of cumulative average abnormal returns, and *T* is the number of rebalancing intervals.

## 3. Fund Ratings in US Mutual Funds Based on the EU-E Decision Model

### 3.1. Data and Descriptive Statistics

We apply the fund rating approach based on the EU-E decision model in US mutual funds, which comprise the largest share of the global industry [50]. To demonstrate a distinct aspect of the fund rating approach based on the EU-E model, we use the same dataset as in Chiew et al. [40]. First, we retrieve monthly return and overall rating data for all US mutual funds over the period of August 1992 to July 2015 from the Morningstar Direct database. Then, we exclude funds, which are not assigned an overall rating or have missing data points over the 23-year period. We also exclude funds without 10 years of data prior to August 2002 to calculate the 10-year EU-E ratings. This results in a final sample of 2159 US mutual funds. Furthermore, to conduct the portfolio performance analysis, we collect monthly returns on the S&P 500 index from August 2002 to July 2015 as a proxy for our benchmark from Bloomberg.

The descriptive statistics of the monthly returns for US mutual funds included from August 2002 to July 2015 are shown in Table 1. The total number of observations, *N*, is defined as the total number of funds at each period in the sample period multiplied by the number of months in the sample period.

As shown in Table 1, we observe that the monthly returns during the overall sample period yield skewness of −0.67 and kurtosis of 11.15, deviating from the assumptions of a normal distribution for fund returns. This is confirmed by the highly significant Jacque-Bera test statistic of 956,805.55. To avoid the improper influence of extreme values among all the returns on the distribution of return series, we winsorized the data at the 1% level and performed the following investigation using the winsorized data.

**Figure 1:**
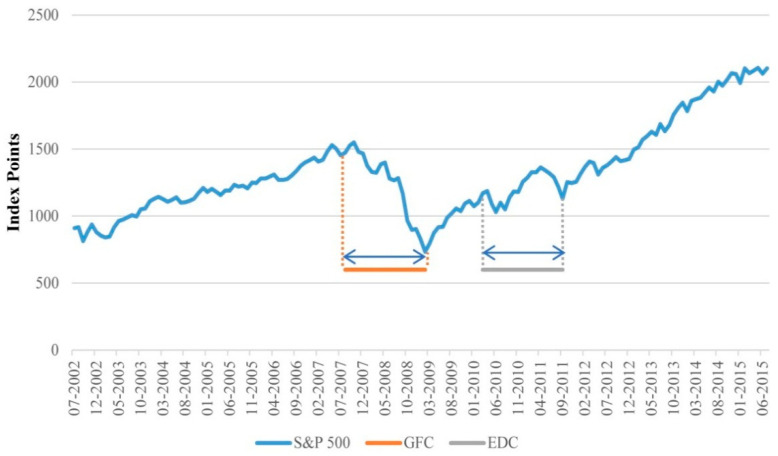
S&P 500 index for 2002-08–2015-07. The date is shown on the horizontal axis and the level of the S&P 500 index, in terms of basis points, on the vertical axis. GFC and EDC denote the global financial crisis and the European debt crisis periods, respectively.

To examine the effect of excessive volatility on the performance of the portfolio, we define subsamples for the global financial crisis (GFC) and the European debt crisis (EDC) periods. The GFC spans the period August 2007 to February 2011, and the EDC comprises the period March 2010 to September 2011. The impact of the EDC on U.S markets was of a smaller magnitude than the GFC as only major events of the EDC sparked uncertainty in the U.S, which hindered the S&P 500′s recovery from the GFC. These events include the Greek bailout in May 2010, the Ireland bailout in November 2010 and speculation of default by major European banks coupled with the rise in the probability of default by Italy and Spain around the third quarter of 2011 [51,52]. The EDC concluded by late 2012 to early 2013, where levels of systemic risk returned to normal levels [51]. Our EDC sample spans the period March 2010 to September 2011, which includes the major events that had a considerable impact on the S&P 500 index. Figure 1 illustrates the performance of the S&P 500 index during the overall sample period.

### 3.2. Stability of Fund Ratings Based on the EU-E Decision Model and Morningstar

We use the selected 2159 US mutual funds as the final sample over the period of August 1992 to July 2015. Then, we calculate the probability distribution of returns for each fund. The monthly return of each fund is between *a* and *b*, where *a* and *b* are the minimum and maximum of all fund returns, respectively. For the 2159 US mutual funds over the period, *a* = −10.53%, *b* = 10.98%. Let $r_{0}=-0.11$ and $r_{11}=0.11$, we divide the monthly return distribution into 11 equal subintervals $\left[r_{0},-0.09\right]$, [−0.09, −0.07), …, [0.07, 0.09), $\left[0.09,r_{11}\right]$, and we denote these subintervals as $\theta_{1}$, $\theta_{2}$, …, $\theta_{11}$, respectively. In this paper, we obtain 276 returns for each fund. Therefore, Bernoulli’s theorem holds. Thus, we can use $\rho_{ij}$ as an estimation of $p_{ij}$. We can then obtain the probability distribution of each sample fund.

Before applying the EU-E model for fund ratings, we need to determine investors’ utility function *u*(*x*). For the sake of simplicity, we suppose u(x)=x. We calculate each of the 2159 funds’ net expected utility by subtracting their calculated risk from the expected utility in the same month. We then assign each of the funds a rank based on their net expected utility, where 5 is assigned to the fund with the highest net expected utility and 1 to the fund with the lowest net expected utility. We also calculate the 3-year ratings at each month for each of the funds. Then, we construct the probability distribution for all funds based on the returns from the previous three years before the selected month (*l* = 3 years or 36 months). Next, we calculate the respective entropy, expected utility and risk values based on the probability distribution above for each fund. We repeat this process for a fund’s past 5- and 10-year returns. Finally, we obtain each fund’s overall rating using Morningstar’s “50:30:20” approach each month, resulting in our monthly fund rankings based on the EU-E model [40].

**Figure 2:**
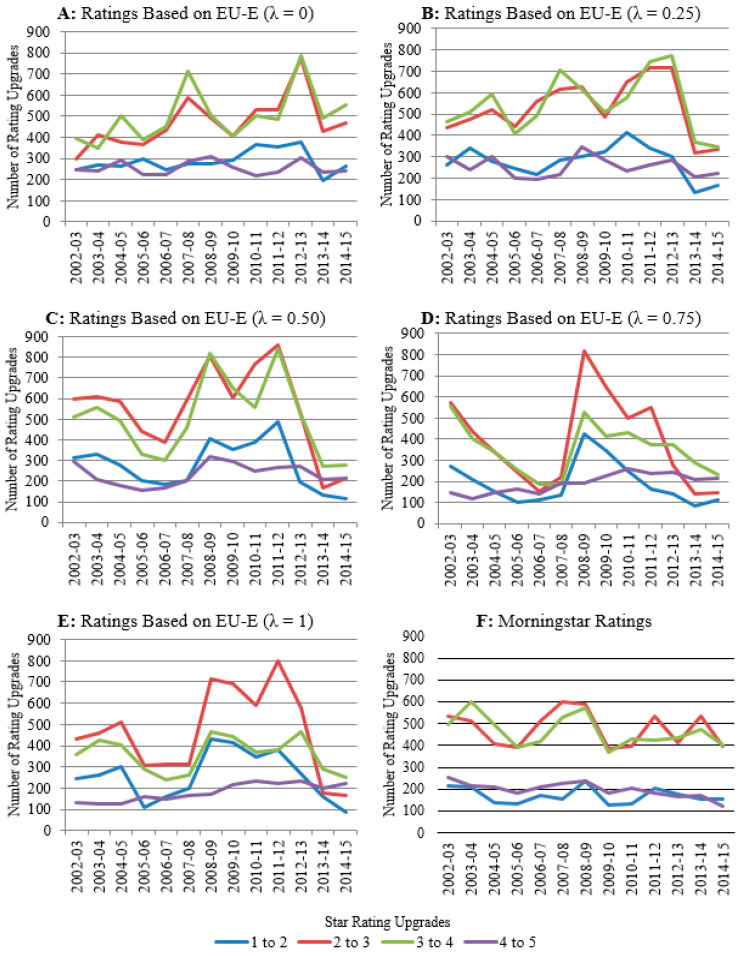
Number of rating upgrades by year. Note: This figure illustrates the number of rating upgrades from lower to higher grades (e.g., from 1 to 2 stars, 2 to 3 stars, etc.) using the ratings based on Morningstar and EU-E (λ = 0), EU-E (λ = 0.25), EU-E (λ = 0.50), EU-E (λ = 0.75), and EU-E (λ = 1).

To explore the differences in the information given by the ratings from the EU-E models and Morningstar, we have conducted different patterns for different λ from 0 to 1. It is not necessary to explain all the results when λ takes values from 0 to 1. Here, we illustrate the results of the fund ratings using the EU-E models when λ = 0, 0.25, 0.5, 0.75, 1. The reason why we only chose these discrete values of λ, for example, why we chose 0.25 and 0.75, is because these were both midpoints when selecting expected utility-driven fund ratings and entropy-driven fund ratings. These values of λ are typical enough to represent fund ratings when λ takes different values from 0 to 1. We plot the number of rating upgrades from lower to higher grades each year in the overall sample using the ratings based on the EU-E model and Morningstar in Figure 2. This figure illustrates the number of rating upgrades using the ratings based on Morningstar and EU-E (λ = 0), EU-E (λ = 0.25), EU-E (λ = 0.50), EU-E (λ = 0.75), and EU-E (λ = 1). The yearly intervals begin in August and end in July of the denoted years. The maximum number of rating s at each yearly interval is 25,908.

Overall, we find that ratings based on the EU-E model capture different information from Morningstar ratings. Furthermore, we also note that the relative weighting of λ in the calculation of risk tends to impact the information provided by the ratings based on the EU-E model. It is also interesting to note that the largest proportion of ratings for all EU-E models occurs within the latter half of the sample period (i.e., 2008 onwards), but for Morningstar, ratings occur during the first half of the sample period.

Panels A and B of Figure 2 illustrate the number of rating upgrades from lower to higher grades of the EU-E (λ = 0) and EU-E (λ = 0.25) models, respectively. The number of upgrades from 1 to 2 stars and 4 to 5 stars remain relatively constant throughout the overall sample period. In contrast, there is a greater number of upgrades from 2 to 3 and 3 to 4 stars. This implies that much of the variation in star ratings from the EU-E (λ = 0) and EU-E (λ = 0.25) models are within the 2- to 4-star categories and that funds with a 1- or 5-star rating tend to persist over time.

Panels C, D and E of Figure 2 show the number of rating upgrades of the EU-E (λ = 0.50), EU-E (λ = 0.75) and EU-E (λ = 1) models, respectively. As opposed to the EU-E (λ = 0) and EU-E (λ = 0.25) models, there is greater volatility within each rating increment. In particular, there is a surge in the number of upgrades for each rating increment at the 2008–2009 interval, which persists until the 2012–2013 interval. This may be explained by the rise in market volatility during the GFC and EDC periods. In the EU-E model, risk comprises both expected utility and entropy. As λ approaches 1 (0), a greater weighting in the measure of risk is placed towards entropy (expected utility) relative to expected utility (entropy). As entropy penalizes for the volatility in a fund’s returns relative to its peers, the rise in the number of rating changes during volatile markets may be explained by the effect of entropy.

Panel F of Figure 2 shows the number of rating upgrades for Morningstar in the overall sample period. Similar to the EU-E (λ = 0) and EU-E (λ = 0.25) models, the majority of rating changes occur within the 2- and 4-star rating groups. However, unlike the volatility observed in the ratings from the EU-E models across all λ values, there is greater stability in Morningstar ratings.

## 4. Performance of Portfolios Constructed Using EU-E Model and Morningstar Ratings

### 4.1. Portfolio Rebalancing Periods

In this section, we construct portfolios using the 5-star funds based on Morningstar ratings and the EU-E (λ = 0.25) and EU-E (λ = 0.75) models, respectively. We use 15 funds in each portfolio. We construct 100 equally weighted portfolios by randomly selecting 15 funds from the 5-star category ranked by Morningstar and EU-E models.

**Figure 3:**
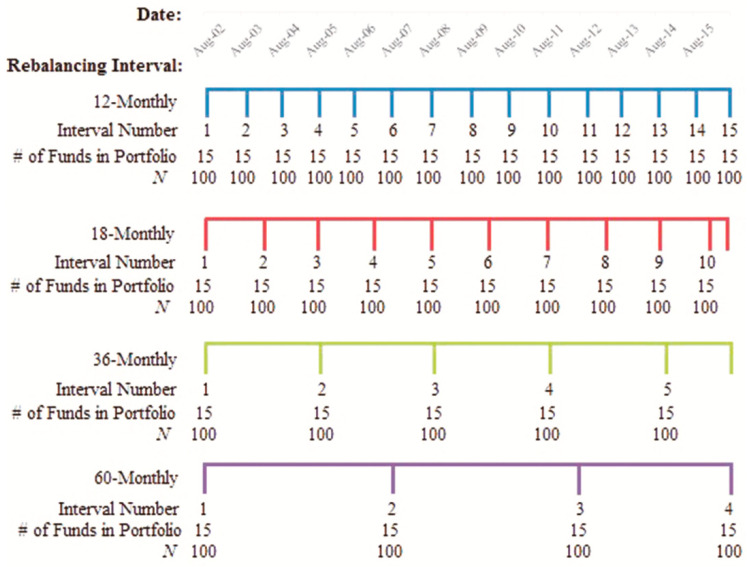
Summary of portfolio rebalancing periods. Each portfolio is rebalanced, and the performance of each portfolio is recorded at each interval, as illustrated by the interval number. *N* refers to the total number of portfolios constructed using each of the ratings based on Morningstar and the EU-E model, where λ takes a value of 0.25 and 0.75.

Figure 3 provides an illustration of the rebalancing interval regimes considered in the analysis of this study. The rebalancing intervals are an important component of this analysis as they are constructed to replicate the choices, which should be made by a typical retail investor. We adopt four different rebalancing intervals as follows: 12-, 18-, 36-, and 60-monthly rebalancing intervals throughout the overall sample period to examine the performance of portfolios formed using ratings based on either Morningstar or the EU-E models.

### 4.2. Abnormal Returns of the Portfolios Based on the EU-E Decision Model and MORNINGSTAR

We use the methods in Section 2.2 to evaluate the performance of portfolios in the following. First, in Table 2, we provide a comparative summary of the performance of 100 randomly selected, equally weighted portfolios constructed using the 5-star categories of the ratings based on the EU-E (λ = 0.25), EU-E (λ = 0.75), and Morningstar. The total number of portfolios constructed for each rating measure within each testing period is 100. The mean, minimum, maximum, and standard deviations are reported in terms of the return of the rating measure relative to the S&P 500. The number of significant outperformances and underperformances relative to the S&P 500 are reported as a percentage of the total number of periods per portfolio (*N*). The significance of the portfolios is tested at the 10% level.

In general, the ratings based on the EU-E (λ = 0.25) provide the best performing portfolios. In each panel, the number of times a portfolio constructed using the ratings based on the EU-E (λ = 0.25) significantly outperforms the benchmark is above 56%. The outperformance statistic is greatest in the 12- and 60-month rebalancing intervals where the proportion is 68.31% and 71%, respectively. Additionally, the number of significant underperformances of the portfolios constructed using the ratings based on the EU-E (λ = 0.25) relative to the benchmark is at a maximum in the 12-month rebalancing interval portfolios at 7% and declines as the rebalancing period increases.

The results are much weaker for the portfolios constructed using the ratings based on the EU-E (λ = 0.75) and Morningstar. The highest proportion in which the portfolios constructed using the 5-star funds of the EU-E (λ = 0.75) and Morningstar significantly outperform the benchmark is 34.8% and 17.62%, respectively. Furthermore, the result supports our expectation that portfolios constructed using the ratings based on the EU-E (λ = 0.75) outperform the Morningstar rating portfolios. The number of times the portfolios constructed using the ratings based on the EU-E (λ = 0.75) outperform the benchmark is proportionally larger than that of Morningstar ratings, while the underperformance statistic of the portfolios based on Morningstar ratings is greater than the number of outperformances at each rebalancing interval. For instance, for the 12-month rebalancing interval, the proportion of Morningstar rating-based portfolios, which significantly underperform the benchmark, is 21.08%, whereas the number of significant outperformance is only 17.62%. For the ratings based on the EU-E (λ = 0.75), the number of portfolios, which significantly outperform the benchmark is 29.77%, whereas the number of significant underperformances is only 8.77%.

The statistics presented on the overall excess returns provide further support for our above-mentioned findings. In each rebalancing interval, the mean excess return on the portfolios using the ratings based on the EU-E (λ = 0.25) is the highest among the three rating models with an excess return of 0.50%, on average across the four rebalancing intervals. This is followed by the portfolios constructed using the 5-star funds of the EU-E (λ = 0.75) and Morningstar, which yield average excess returns of 0.21% and −0.03%, respectively, across the four rebalancing intervals. For the portfolios based on Morningstar ratings, the mean excess return is only positive for the 12-month rebalancing interval returning 0.06%. The standard deviation of excess returns is consistent across the rebalancing periods for each model.

In general, we find that the performance of portfolios based on the ratings of both EU-E (λ = 0.25) and EU-E (λ = 0.75) models tend to improve over longer rebalancing intervals. In contrast, portfolios constructed using Morningstar ratings decreases as the length of the rebalancing interval increases.

### 4.3. Average Abnormal Returns of the Portfolios Based on the EU-E Decision Model and Morningstar

This section illustrates the average abnormal returns across the 100 equally weighted portfolios based on the 5-star funds ranked by the EU-E models (λ = 0.25 and 0.75) and Morningstar at every rebalancing interval across the overall sample period.

**Figure 4:**
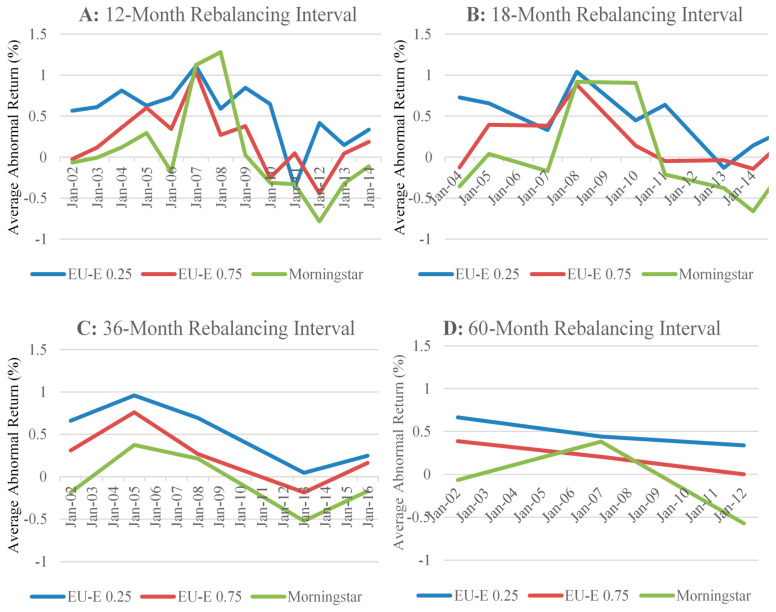
Average abnormal returns of portfolios through time. The abnormal return of the rating measure relative to the benchmark as a % is illustrated on the vertical axis and the date on the horizontal axis.

Consistent with the findings reported in Table 2, the portfolios constructed using the ratings based on the EU-E (λ = 0.25) remain the best performers. This result is complemented by Panel A of Figure 4, where the portfolios using the 5-star funds based on the EU-E (λ = 0.25) consistently earn positive abnormal returns in the 12-month rebalancing intervals, a result which is statistically significant at the 10% level. These portfolios only underperform the S&P 500 in the interval between August 2011–July 2012, where the proportion of portfolios record a statistically 62% significant underperformance. The consistent outperformance relative to the portfolios benchmark based on the EU-E (λ = 0.25) across varying market climates highlights the ability for the EU-E model to effectively capture investor preferences, as shown by Yang and Qiu [22].

**Figure 5:**
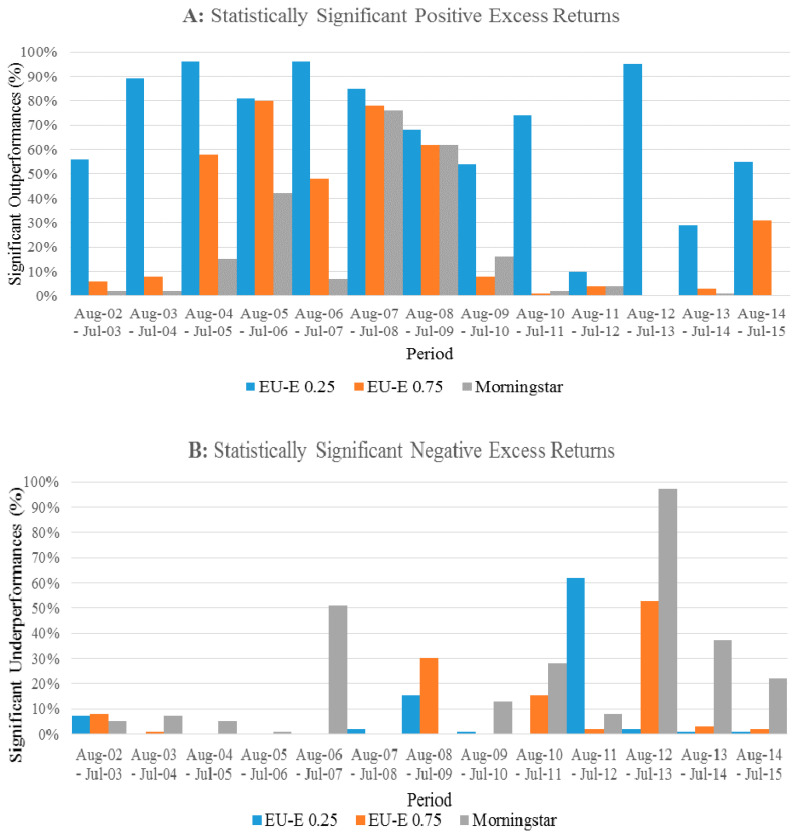
Summary of statistically significant abnormal returns over 12-month rebalancing intervals. This figure shows the 12-month interval on the horizontal axis and the number of statistically significant outperformances as a percentage on the vertical axis. Statistical significance is tested at the 10% level.

Panel A of Figure 4 shows that the portfolios based on Morningstar ratings perform particularly well, earning an average excess return relative to the S&P 500 of 1.13% and 1.27% in the periods to July 2008 and July 2009, respectively. This is compared to the fall in excess return by the ratings based on the EU-E (λ = 0.25) and EU-E (λ = 0.75) of 1.11% to 0.59% and 1.04% to 0.27%, respectively over the same period. The abnormal return earned by the portfolios constructed using Morningstar ratings during this period does not persist. This result is further complemented by Figure 5, where at only three intervals do the Morningstar ratings portfolios record a notable statistically significant and positive excess return: August 2005–July 2006 (42%), August 2007–July 2008 (76%), and August 2008–July 2009 (62%). In contrast, the proportion of portfolios based on Morningstar ratings, which earn negative and significant excess returns, is only recognizable in the August 2006–July 2007 (51%) and August 2012–July 2013 (97%) intervals. At other times, no significant difference from the return of the S&P 500 is recorded. This finding demonstrates that the 5-star category of Morningstar ratings is unable to select the best performing funds consistently.

As illustrated in Panels C and D of Figure 4, the portfolios performance based on the ratings calculated using EU-E (λ = 0.75) tend to lie between the portfolios constructed using the ratings based on the EU-E (λ = 0.25) and Morningstar ratings. In addition, Panel A of Figure 5 demonstrates that the proportion of portfolios constructed using the ratings based on the EU-E (λ = 0.75), which earn positive and significant excess returns exceeds 50% in the four 12-month rebalancing intervals beginning August 2004 to August 2008. The proportion of portfolios, which earn negative and significant excess returns is only in excess of 50% in the August 2012–July 2013 interval at 55%. Like Morningstar ratings, the portfolios constructed using the 5-star funds of the EU-E (λ = 0.75) model are generally unable to differentiate their performance from the S&P 500 at most rebalancing intervals. Thus, a possible explanation for the similarity in the portfolios performance constructed using the 5-star funds of the EU-E (λ = 0.75) and Morningstar is that these models are unable to efficiently capture the risk faced by investors.

Overall, the differentiation between each of the three models is evident in their relative performance. From the results presented in Figure 4 and Figure 5, the portfolios constructed using the ratings based on the EU-E (λ = 0.25) provide the best performance across each rebalancing interval. Although the portfolios using the ratings based on the EU-E (λ = 0.75) outperform Morningstar, we show that the performance of the two models is relatively similar. Notably, we find that the risk measure of each model plays a significant role in the performance of the portfolios. Consistent with Yang and Qiu [22], we show that the EU-E model produces outcomes, which consider an investor’s behavior under risk. This is demonstrated by the persistent positive and significant abnormal returns earned by the portfolios constructed using the 5-star funds of the EU-E (λ = 0.25) model over the 13-year sample period. In contrast, we further prove a higher λ value (i.e., 0.75) to be less efficient than a lower one (i.e., 0.25). This is because a higher λ value may overweight the effect of entropy in measuring risk during stable market conditions. As such, we find a relatively indistinguishable performance by the portfolios constructed using the 5-star funds of the EU-E (λ = 0.75) model relative to the S&P 500. Similarly, we find evidence consistent with Lisi and Caporin [6] that the constant relative risk aversion coefficient in calculating Morningstar ratings is inefficient in capturing the risk preferences of investors.

### 4.4. Cumulative Average Abnormal Returns of the Portfolios Based on EU-E Decision Model and Morningstar

This section assesses the ability of an investor to construct trading strategies using the ratings based on the EU-E model and Morningstar by examining the cumulative average abnormal returns earned by the portfolios across rebalancing intervals.

**Figure 6:**
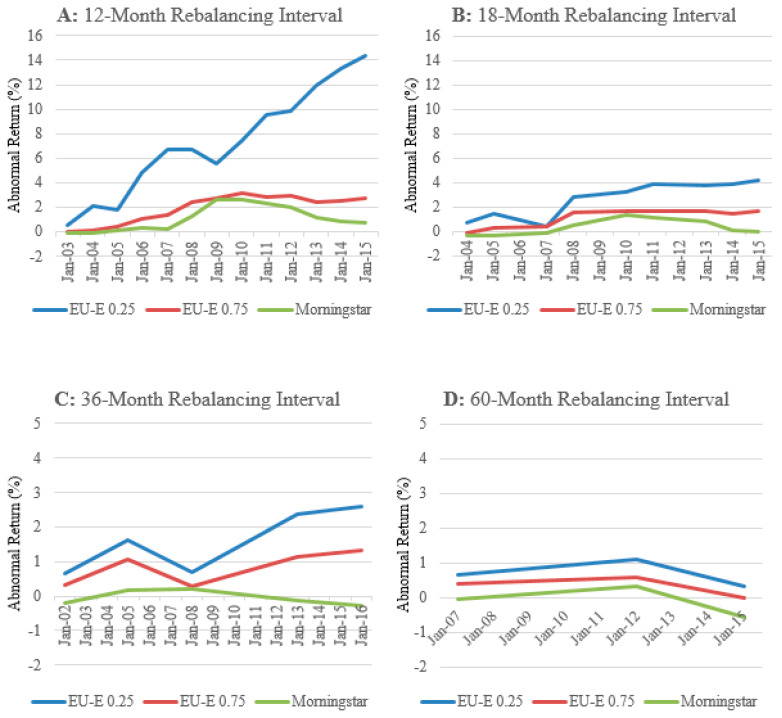
Cumulative average abnormal returns during the sample period. The vertical axis shows the cumulative average abnormal return of the portfolios constructed using the rating models relative to the benchmark as a percentage.

Figure 6 illustrates the cumulative average abnormal returns earned by the 100 portfolios constructed using the ratings based on the EU-E (λ = 0.25), EU-E (λ = 0.75) and Morningstar, for the 12-, 18-, 36-, and 60-month rebalancing intervals throughout the sample period.

Panel A of Figure 6 shows the cumulative average abnormal returns generated by the 100 portfolios constructed using 5-star funds from the EU-E (λ = 0.25), EU-E (λ = 0.75) and Morningstar, for the 12-month rebalancing interval. Consistent with our previous findings, the portfolios constructed using the ratings based on the EU-E (λ = 0.25) earn the greatest abnormal returns of the three models, earning an economically and statistically significant cumulative average abnormal returns of 14.35% throughout the 13-year sample period. This is compared to the cumulative average abnormal returns of 2.67% produced by the portfolios constructed using the ratings based on the EU-E (λ = 0.75), and 0.74% by the portfolios constructed using Morningstar ratings in the 12-month rebalancing intervals over the period August 2002 to July 2015. The cumulative average abnormal returns earned by the portfolios constructed using the ratings based on the EU-E (λ = 0.75) and Morningstar are economically insignificant and highlight our finding that these two models are unable todifferentiate their performance from the benchmark due to inefficiencies in capturing risk.

Furthermore, we report the cumulative average abnormal returns of the portfolios based on 5-star funds from the EU-E (λ = 0.25), EU-E (λ = 0.75) and Morningstar models for the 12-, 18-, 36-, and 60-monthly rebalancing intervals at the end of the sample period.

We find that the significance, both economic and statistical, of the cumulative average abnormal return for each model decreases as the length of the rebalancing interval increases. Panel B of Figure 6 shows the cumulative average abnormal return for each model during the overall sample period using 18-month rebalancing intervals. For the portfolios constructed using the ratings based on the EU-E (λ = 0.25), the cumulative average abnormal return falls to 4.18%. Likewise, the cumulative average abnormal return earned by the portfolios constructed using the ratings based on the EU-E (λ = 0.75) and Morningstar declines to 1.64% and −0.07%, respectively. Similar declines for each rating model are observed in Panels C and D of Figure 6, which illustrate the 36- and 60-month rebalancing intervals, respectively.

Overall, we can conclude from the results presented in Figure 6 and Table 3 that a shorter rebalancing interval (i.e., 12-monthly) is relatively more optimal for an investor who wishes to maximize the return on their portfolio. Additionally, the findings presented show that only the portfolios constructed using the ratings based on the EU-E (λ = 0.25) model produce economically significant abnormal returns for an investor. Therefore, the cumulative average abnormal return of portfolios constructed using 5-star funds from the EU-E (λ = 0.25) model relative to Morningstar lends further support that a portfolio constructed using 5-star funds from the EU-E model outperforms that of Morningstar.

## 5. Conclusions

We recently established the EU-E fund rating approach and showed that the fund rating approach could predict the best performing funds compared to Morningstar ratings. We applied the approach to the mutual fund ratings in the US market. In this paper, we investigate the practical applicability that investors utilize this fund rating approach to guide their investment decisions. The results in this paper indicate that the fund rating approach based on the EU-E model can assist investors in constructing portfolios of mutual funds and that a portfolio constructed using best ranking funds from the EU-E model outperforms that of Morningstar. This finding is significant as it proves that the EU-E model is relevant in the decision-making process for investors.

Specifically, we construct portfolios based on the best-rated funds to examine the ability for investors to use the ratings in fund selection. Specifically, we construct 100 equally weighted portfolios by randomly selecting 15 funds from the 5-star category ranked both by fund rating based on the EU-E models (λ = 0.25, λ = 0.75) and Morningstar. Overall, we find that the portfolios based on the EU-E (λ = 0.25) model perform the best, followed by portfolios using the ratings based on the EU-E (λ = 0.75) model and Morningstar, respectively. Furthermore, only the portfolios based on ratings using the EU-E (λ = 0.25) model produce economically significant CAARs for an investor. We adopt four different rebalancing intervals as follows: 12-, 18-, 36-, and 60-monthly. These findings are robust to these rebalancing intervals.

Our findings in this paper follow on from the finding that the EU-E model can outperform Morningstar ratings in predicting the best performing funds by applying the model in portfolio construction. One future extension to our paper may be to investigate the performance of the EU-E model using shorter time frames. We use a single dataset in this paper, which spans a wide 13 year time period. We adjusted this entire dataset for the age bias as in Morey [53], which may have limited the fund universe considered to seasoned funds only. If the dataset is adjusted for the age bias in smaller time frames, the fund universe considered may be greater and incorporate younger funds.

In addition, DeMiguel et al. [54] evaluate the out-of-sample portfolio performance based on 14 models in terms of Sharpe ratio, certainty-equivalent returns and turnover. The portfolio performance is evaluated based on pure abnormal returns. In addition, DeMiguel and Nogales [55] propose a class of portfolios that have better robust properties than the traditional minimum variance portfolios. Thus, it is quite interesting for us to further investigate portfolio performance using the above measures and future approaches.

## Figures and Tables

**Table 1:** Descriptive statistics of the monthly returns of US mutual funds.

*N*	Mean	S.D.	Skewness	Kurtosis	Min.	Med.	Max.	Jacque-Bera
336,804	0.59	3.61	−0.67	11.15	−46.20	0.58	34.09	956,805.55

**Table 2:** Summary of AAR of portfolio performance.

	Mean	Std. Dev.	Min.	Max.	Significant Outperformance (%)	Significant Underperformance (%)	*N*
**Panel A:** 12 Month Rebalancing Interval							
EU-E (λ = 0.25)	0.54	0.55	−2.18	2.44	68.31	7.00	1300
EU-E (λ = 0.75)	0.21	0.48	−1.79	2.31	29.77	8.77	1300
Morningstar	0.06	0.62	−1.55	2.32	17.62	21.08	1300
**Panel B:** 18 Month Rebalancing Interval							
EU-E (λ = 0.25)	0.46	0.54	−1.19	2.42	56.89	5.44	900
EU-E (λ = 0.75)	0.18	0.45	−1.44	1.96	32.11	10.00	900
Morningstar	−0.01	0.59	−1.15	1.79	10.67	21.44	900
**Panel C:** 36-Month Rebalancing Interval							
EU-E (λ = 0.25)	0.52	0.54	−1.22	2.29	56.40	4.40	500
EU-E (λ = 0.75)	0.26	0.45	−0.81	1.66	34.80	7.00	500
Morningstar	−0.06	0.41	−1.21	1.12	4.40	17.60	500
**Panel D:** 60-Month Rebalancing Interval							
EU-E (λ = 0.25)	0.48	0.47	−1.14	1.65	71.00	3.67	300
EU-E (λ = 0.75)	0.20	0.37	−0.75	1.31	25.33	8.33	300
Morningstar	−0.09	0.46	−0.98	1.25	5.67	30.33	300

Note: Table 2 summarizes the average abnormal returns (AAR) of portfolios constructed using the ratings based on the EU-E model and Morningstar across 12-, 18-, 36- and 60-month rebalancing intervals.

**Table 3:** Cumulative average abnormal returns of portfolios based on 5-star funds from the EU-E model and Morningstar.

Model	Rebalancing Interval			
	12-Monthly	18-Monthly	36-Monthly	60-Monthly
EU-E (λ = 0.25)	14.35 ***	4.18 ***	2.60	0.34
	(11.19)	(3.15)	(1.33)	(0.12)
EU-E (λ = 0.75)	2.67 ***	1.64	1.31	0.00
	(2.82)	(1.54)	(0.90)	(−0.01)
Morningstar	0.74	−0.07	−0.29	−0.57
	(1.01)	(−0.08)	(−0.26)	(−0.43)

Note: The cumulative average abnormal return is reported as a percentage (%). *T*-statistics are in parenthesis. *** indicates statistical significance at the 1% levels.

## Data Availability

The data presented in this study are openly available from the Morningstar Direct database and from Bloomberg, as shown in Section 3.1.
